# IL-17A Signaling in Colonic Epithelial Cells Inhibits Pro-Inflammatory Cytokine Production by Enhancing the Activity of ERK and PI3K

**DOI:** 10.1371/journal.pone.0089714

**Published:** 2014-02-25

**Authors:** Xiaoqin Guo, Xingwei Jiang, Yan Xiao, Tingting Zhou, Yueling Guo, Renxi Wang, Zhi Zhao, He Xiao, Chunmei Hou, Lingyun Ma, Yanhua Lin, Xiaoling Lang, Jiannan Feng, Guojiang Chen, Beifen Shen, Gencheng Han, Yan Li

**Affiliations:** 1 Department of Molecular Immunology, Beijing Institute of Basic Medical Sciences, Beijing, China; 2 Department of Immunology, Logistics College of Chinese People’s Armed Police Forces, Tianjin, China; 3 Department of Respiratory Medicine, First Affiliated Hospital of the Chinese PLA General Hospital, Beijing, China; 4 Department of Medical Engineering, Military General Hospital of Beijing PLA, Beijing, China; Massachusetts General Hospital, United States of America

## Abstract

Our previous data suggested that IL-17A contributes to the inhibition of Th1 cell function in the gut. However, the underlying mechanisms remain unclear. Here we demonstrate that IL-17A signaling in colonic epithelial cells (CECs) increases TNF-α-induced PI3K-AKT and ERK phosphorylation and inhibits TNF-α induced expression of IL-12P35 and of a Th1 cell chemokine, CXCL11 at mRNA level. In a co-culture system using HT-29 cells and PBMCs, IL-17A inhibited TNF-ãinduced IL-12P35 expression by HT-29 cells and led to decreased expression of IFN-γ and T-bet by PBMCs. Finally, adoptive transfer of CECs from mice with Crohn's Disease (CD) led to an enhanced Th1 cell response and exacerbated colitis in CD mouse recipients. The pathogenic effect of CECs derived from CD mice was reversed by co-administration of recombinant IL-17A. Our data demonstrate a new IL-17A-mediated regulatory mechanism in CD. A better understanding of this pathway might shed new light on the pathogenesis of CD.

## Introduction

Crohn's disease (CD) and ulcerative colitis (UC) are two forms of inflammatory bowel disease (IBD) in man. The etiology of IBD remains unclear, but evidence indicates that it results from an interaction between genetic and environmental factors, which eventually lead to an excessive and poorly controlled mucosal inflammatory response directed against components of the normal microflora and mucosal constituents of the gut [1]–[2]. Studies over the last 2–3 decades have shown that different T cell differentiation patterns determine disease progression [3]–[4]. For example, it is known that CD is linked to a predominantly T helper cell (Th1) immune response (e.g., secretion of IFN-γ, TNF-α, and IL-12). Accordingly, therapeutic strategies targeting these cytokines have been widely investigated. Antibody against TNF-α attenuates colitis in IBD patients, but more than one third of IBD patients do not respond to anti-TNF-α therapy [5]-[6]. These observations suggest the need to identify novel targets for therapeutic intervention in IBD**.**

In addition to the classical Th1/Th2 pathways, a new pathway, the Th17 pathway, has been discovered as a result of the identification of a novel CD4 T cell subset, the Th17 cell [7]. It is now known that IL-17A has pro-inflammatory effects on a wide range of cellular targets, such as epithelium, endothelium, and monocytes/macrophages [8]–[10], and plays pathogenic roles in some organ-specific autoimmune diseases, such as rheumatoid arthritis (RA) and multiple sclerosis, as well as IBD [11]. Because of this, the therapeutic effects of an IL-17 neutralizing antibody, secukinumab (AIN457T), in RA are now being evaluated in phase II clinical trials [12]. As regards IBD, IL-17A is produced in the healthy gut, but high IL-17A mRNA expression is seen in inflamed colonic mucosa [13]-[14], suggesting a pathogenic role of IL-17A in the progression of IBD. Accordingly, IL-17A has been examined as a target for reducing autoimmune damage in IBD [15]. Unfortunately, clinical trials targeting IL-17A in IBD failed to show an effect, indicating that further studies are needed on its role in IBD.

It is now known that there is a complex and active interplay between IL-17A and colonic epithelial cells (CECs) during the progression of IBD. After stimulation by IL-17A, CECs release a wide range of pro-inflammatory cytokines and chemokines, e.g., CXCL8 for neutrophil chemotaxis and CCL20 to attract Th17 cells, further amplifying the gut inflammation [16]. On the other hand, IL-17A also has protective effects on the gut epithelial barrier, e.g., by upregulating the expression of antimicrobial peptides [17]. Recent data have also shown that IL-17A, by directly binding to its receptor (IL-17R) expressed on Th1 cells, inhibits Th1 cell-mediated colonic inflammation [18].Together, these data suggest that IL-17A plays both a pro-inflammatory and an anti-inflammatory role in IBD, which might explain the failure of the clinical trial targeting IL-17A. To explore more effective intervention strategies, the mechanisms by which IL-17A mediates its pathogenic or protective effects, especially the latter, need to be investigated.

In most target cells, IL-17A signaling activates the MAPK and NF-κB pathways through IL-17RA and increases the expression of inflammatory cytokines [16]. Act1 has been identified as an essential adaptor molecule in IL-17 signaling [19]. In addition, the results of a microarray screen suggested the involvement of the CCAAT/enhancer binding protein transcription factors C/EBPβ and C/EBPδ in the IL-17A-induced signaling cascade [20], while another report showed that the PI3K pathway is involved in IL-17A signaling, mainly in an Act1-independent manner [21], but the underlying mechanisms remain largely unclear. Further investigation of the signaling mechanisms of IL-17A will shed light on its biological functions and help in understanding and treating inflammatory diseases.

Our previous data suggested that IL-17A signaling inhibited the function of Th1 cell in IBD [22]. However, the underlying mechanisms remain largely unclear. Although some data suggest that IL-17A suppresses the development of colonic inflammation by directly inhibiting the differentiation of Th1 cells [18], we argue that other mechanisms may exist, since IL-17A binds to multiple target cells and stimulates complex intracellular cascades. In this study, CECs were used as the target for IL-17A and we demonstrated, for the first time, that IL-17A signaling in CECs can also trigger anti-inflammatory mechanisms by activating the PI3K-AKT and ERK-CEBP/β pathways in an Act1-dependent manner, finally leading to inhibition of TNF-α-induced expression of IL-12P35 and of a Th1 cell chemokine, CXCL11, and of Th1 cell function. This is the first report demonstrating the involvement of the Act1-PI3K-AKT pathway in the IL-17A-triggered signaling cascade. Further investigation of this pathway should shed new light on therapeutic strategies against many IL-17A–related clinical diseases

## Materials and Methods

### Cell culture and gene expression

HT-29 human colorectal cancer cells (ATCC) were cultured in McCoy’s 5A medium (ATCC) supplemented with 10% fetal bovine serum (FBS), penicillin (10 U/ml), and streptomycin (10 µg/ml) (all from Sigma-Aldrich). For tests, they were plated in 12-well plates at a density of 3×10^5^ cells per well in McCoy’s 5A medium containing 10% FBS and antibiotics. Before cytokine treatment, the cells were incubated overnight in McCoy’s 5A medium containing 0.5% FBS and antibiotics, then were incubated for 6 h with different dose of TNF-α (R & D Systems) and/or of IL-17 (eBiosciences, San Diego, CA). Here 0.5 ng/ml of TNF-α (suboptimal dose from which we can see the effects of IL-17A) and/or 50 ng/ml of IL-17 were used for in vitro cell stimulation. The cells were then harvested and RNA prepared using Trizol reagent (Invitrogen). RNA samples (2 µg) were then reverse-transcribed with Moloney murine leukemia virus reverse transcriptase (New England Biolabs) and real-time PCR performed using SYBR Green (TOYOBO) and a standard curve for quantization, as described previously [23].

The relative expression of cytokine mRNAs was evaluated by real-time PCR. The real-time PCR reaction mixture consisted of 10 µl of 2×SYBR green Master Mix, 0.5 µl of 10 pM primers, and 2 µl of cDNA in a total volume of 20 µl. The thermal cycling conditions were an initial denaturation step at 95°C for 3 min; 40 cycles at 95°C for 10 s, annealing at 60°C for 15 s, and extension at 72°C for 10 s; and 71 cycles at 60°C for 30s. The sequences of the primers used, produced by Assays-by-Design Service for Gene Expression Assays (Biomics Biotechnologies Co. Ltd., China), are listed in Table 1. At the end of the PCR cycles, the specificity of the amplification products was checked by dissociation curve analysis. mRNA levels in each sample were determined using the gene-specific threshold cycle (Ct) for each sample (△Ct) corrected by subtracting the Ct for the GAPDH housekeeping gene. Untreated controls were used as the reference samples and the △Ct for all experimental samples was subtracted from the △Ct for the control samples (△△Ct). The magnitude of the change in levels of the test gene mRNA was expressed as 2-△△Ct. Each measurement was performed in duplicate.

**Table 1:** Sequences of the primers used for real-time PCR.

	Forward primer	Reverse primer
hCXCL11	GAGGACGCTGTCTTTG	GATTTGGGATTTAGGC
hIL-12P35	ACCACTCCCAAAACCTGC	CCAGGCAACTCCCATTAG
hAct1	AACAAGGAAGCATGAATTTCAGA	ATTCTTGGGCCAGCTGTAGA
hPI3K-CG	CTGGAAAGAAGACAAGCCCA	TTAACTGGGGCATTCCTGTC
hIFN-γ	ACTGACTTGAATGTCCAACGCA	ATCTGACTCCTTTTTCGCTTCC
hT-bet	CCACCTGTTGTGGTCCAAGT	AACATCCAGTAGTGGCTGGTG
hCCL20	CTGGCTGCTTTGATGTCAGT	CGTGTGAAGCCCACAATAAA
hGAPDH	AACGGATTTGGTCGTATTG	GGAAGATGGTGATGGGATT
mIFN-γ	AAGCGTCATTGAATCACACCTG	TGACCTCAAACTTGGCAATACTC
mIL-12a	CGCAGCACTTCAGAATCACA	TCTCCCACAGGAGGTTTCTG
mCXCL11	AAGGTCACAGCCATAGCCCT	CATTTTGACGGCTTTCATC
mCCL20	CCAAGTCTTCTCAGCGCCAT	GAATCTTCCGGCTGTAGGAGAAG
mGAPDH	TCTTGGGCTACACTGAGGAC	CATACCAGGAAATGAGCTTGA

h indicates human and m mouse.

### Western blots

Western blotting was performed to evaluate levels of ERK, AKT, phospho-ERK, phospho-AKT, phospho-C/EBPβ, PI3K p110γ, Act1, and GAPDH. Briefly, 30 ug of protein was electrophoretically separated on a 12% sodium dodecyl sulfate-polyacrylamide gel and transferred to a polyvinylidene difluoride membrane, which was then blocked by incubation for 1 h at room temperature in 5% fat-free dry milk in Tris-buffered saline containing 0.1% Tween 20 (TBST). The blots were then incubated overnight at 4°C with rabbit antibodies against human ERK (1∶1000), AKT (1∶1000), phospho-ERK (1∶1000), phospho-AKT (1∶1000), phospho-C/EBP(1∶1000), or PI3K p110γ(1∶1000) (Cell Signaling Technology, USA), rat antibodies against human Act1 (eBiosciences, San Diego, CA), or mouse antibodies against GAPDH (1:5000) (Tianjin Sungene Biotech Co. Ltd) diluted in TBST containing 5% BSA, washed for 25 min with TBST, and incubated for 1 h at room temperature with alkaline phosphatase-conjugated anti-rabbit, anti-mouse, or anti-rat IgG antibodies (KPL, Gaithersburg, MD, USA) (1∶2000 in TBST containing 5% BSA).

### Act1 gene knockdown in the HT-29 cell line

To directly examine whether Act1 was involved in the IL-17 signaling pathway, Act1 gene expression in HT-29 cells was blocked using short-hair RNA (shRNA). Three non-overlapping shRNA duplexes (Biomics Biotechnologies Co. Ltd, China) were individually tested for maximal knockdown of gene expression. The duplex sequences were CCATAGACACGGGATATGA (shRNA1), CCCTGAAACTTGCAAATC A (shRNA2), CTGCAATTGACATATTTGA (shRNA3), and TTCTCCGAACGTGTCACGT. (negative control (NC)). These sequences were inserted into the pRNAT-U6.1/Neo vector, then the purified recombinant vectors were transfected into HT-29 cells using Lipofectamine 2000™ (Invitrogen) according to the manufacturer's protocol. The shRNA duplex giving maximal knockdown was identified and HT-29 cell clones stably express Act1 shRNA selected using G418 (Gibco) and analyzed for Act1 expression by Western blotting and RT–PCR.

### Co-culture of peripheral blood mononuclear cells and HT-29 colonic epithelial cells

HT-29 cells were plated in 24-well plates at a density of 1.5×10^5^ cells/well in McCoy’s 5A medium containing 10% FBS and antibiotics and incubated for 24 h, then were treated with IL-17 (50 ng/ml; eBiosciences) and/or TNF- α(0.5 ng/ml; eBiosciences) for 24 h. Human peripheral blood mononuclear cells (PBMCs) were isolated by density gradient centrifugation and added to the culture in a ratio of 1 HT-29 cells to 10 PBMCs. The co-cultures were then stimulated for 24 h by a combination of monoclonal antibodies (mAbs) against CD3 (3 µg/ml) and CD28 (3 µg/ml) ( eBiosciences) with or without IL-12 (12.5 ng/ml; eBiosciences), then non-adherent PBMCs and adherent HT-29 cells were harvested separately for analysis. The human PBMC used in this study have been described in our previous publication [22], and the study protocol was approved by the Ethics Committee of the General Hospital of the Air Force of the PLA, Beijing, China.

### Induction of colitis in mice

Balb/C mice were originally obtained from the Jackson Laboratory, and bred in our facilities under specific pathogen-free conditions. The care, use, and treatment of mice in this study were in strict compliance with the guidelines for the care and use of laboratory animals of the Institute of Basic Medical Sciences, Beijing. The protocol was approved by the Committee on the Ethics of Animal Experiments of the Beijing Institute of Basic Medical Sciences (Permit Number: AMMS2012-0136). All surgery was performed under sodium pentobarbital anesthesia, and all efforts were made to minimize suffering. To induce colitis, 6- 8-week-old male mice were intrarectally injected with 0.2 mg of the hapten reagent 2, 4, 6-trinitrobenzene sulfonic acid (TNBS) (Sigma) in 50% ethanol as previously described [24]–[25]. In control experiments, mice received 50% ethanol alone. The total injection volume was 100 µI in both groups.

### Anti-IL-17A antibody injection

To test the effect of anti-IL-17A antibody on TNBS--induced colitis, mice were injected intraperitoneally with 100 µg of anti-IL-17 mAb or the same amount of same isotype IgG (Tianjin Sungene Biotech Co. Ltd) on days 1, 3, 5, and 7, and the mice were weighed daily and checked for tissue injury.

### Cell isolation and adoptive transfer

The isolation procedure for the mouse colonic epithelial cells (CEC) and colonic lymphocytes in this study has been described previously [26]. In brief, the muscle layer of the mouse colon was removed with forceps and the whole colon opened longitudinally and cut into sections approximately 0.5 cm long, which were then placed in a 150 ml conical flask containing 20 ml of 15 mM HEPES, 5 mM EDTA, 10% FBS, and 100 µg/ml of gentamycin and incubated at 37°C with shaking for 30 min. The sample was then filtered at room temperature through a 200 mesh filter, then the filtrates from three collections were combined and centrifuged at 850 g for 10 min at 37°C and the pellets (CECs) resuspended in phosphate-buffered saline (PBS). For the collection of lymphocytes from colonic lamina propria, colon tissue removed of CECs was further incubated with collagenase D (Roche) (0.6 mg/ml) in 20 ml RPMI-1640 medium at 37°C for about 3 hours. Finally, samples were filtered at room temperature through a 200 mesh filter, then the filtrates from three collections were combined and centrifuged at 850 g for 10 min at 37°C and the pellets (lymphocytes) resuspended in phosphate-buffered saline (PBS). For transfer assay, CECs (1×10^6^ cells/mouse) from TNBS-induced colitis or control mice isolated on day 8 of TNBS treatment were injected into the peritoneum of previously untreated mice on day 1 of TNBS induction of colitis and again on day 4, then the mice were sacrificed on day 8. To test the in vivo effect of IL-17A on the activity of transferred CECs from these TNBS-induced colitis mice were injected intraperitoneally with mouse recombinant IL-17 (eBiosciences, San Diego, CA) at a dose of 500 ng/mouse on days 1,3,5 and 7 of induction of TNBS-colitis.

### Flow cytometry

For staining for IL-17RA, CECs were collected from TNBS-induced colitis mice or control mice, and then were stained with phycoerythrin (PE)-conjugated anti-mouse IL-17RA antibodies (Biolegends). For staining IFN-r within CD4^+^T cells and IL-12 within monocytes/macrophage, cells were stimulated for 4 h with 50 ng/ml of phorbol 12-myristate 13-acetate, 1 µg/ml of ionomycin, and 1 µg/ml of brefeldin A (Sigma, St Louis, MO), then were washed and stained with fluorescein isothiocyanate (FITC)-conjugated anti-human CD4, anti-mouse CD4, anti-human CD14 or anti-mouse CD11b, then fixed for overnight with Fix/Perm buffer, washed with permeabilization buffer, stained for 30 min at 4°C with PE-conjugated anti-human IFN-γ, anti-mouse IFN-γ, anti-human IL-12P70 and anti-mouse IL-P70 antibodies(all from eBioscience) and analyzed on a FACScalibur flow cytometer.

### Histopathological analysis

For histopathological analysis, a specimen from the middle part of the colon was fixed in 10% phosphate-buffered formalin, embedded in paraffin, and sectioned and the sections stained with hematoxylin-eosin (H&E).

### Mouse whole colon cultures

Colon tissue (200–300 mg) was washed in cold PBS containing penicillin and streptomycin and cut into small pieces (0.5×0.5 cm), which were cultured (three pieces per mouse) in 24-well flat bottom culture plates in serum-free RPMI 1640 medium (Gibco) at 37°C for 24 h. The culture supernatants were then centrifuged at 9000 g at 4°C for 5 min and stored at –80°C until use.

### ELISA

The concentration of IFN-γ and IL-12P70 in mouse serum was measured using a sandwich ELISA according to the manufacturer’s protocol (eBiosciences, San Diego, CA).

### Statistical analysis

All data are presented as the mean±SD. Statistical analysis was performed using one way or two-way ANOVA. *p* values less than 0.05 were considered significant.

## Results

### IL-17A signaling in human HT-29 colonic epithelia cells inhibits TNF-α-induced expression of CXCL11 and IL-12P35 mRNA by enhancing phosphorylation of AKT, ERK, and CEBP/β

**Figure 1:**
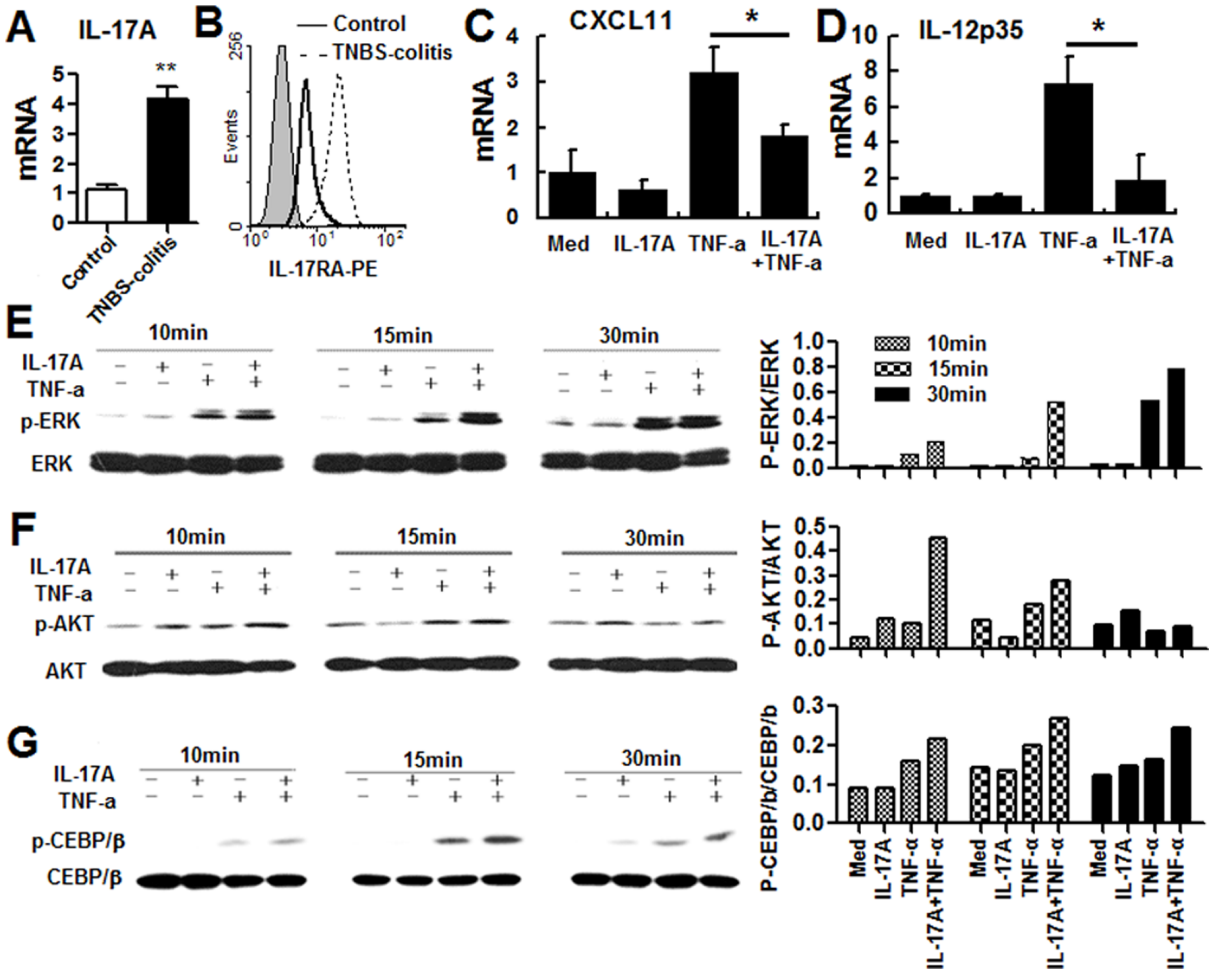
Effects of IL-17A signaling on TNF-α-induced HT-29 cell activation and the intracellular mechanisms. (A&B) CECs were collected from mice as described in the material and methods, and then expressions of IL-17A in and IL-17RA on CECs were examined using real time-PCR(A) or Flow cytometry analysis(B). (C and D) HT-29 cells were stimulated with recombinant IL-17A and/or TNF-a for 6h, then CXCL11 (C) and IL-12P35 (D) mRNA levels were examined by real-time PCR. (E-G) HT-29 cells were treated as above, but for 10 to 30 min, then were examined for the phosphorylation of ERK (E), PI3K-AKT (G), or CEBP/β (G). Band intensity data were shown as well**.** The results shown are representative of those obtained in three independent experiments.

We previously found that levels of IL-17A mRNA and protein are increased and Th1 cell function decreased in patients with IBD [22]. In the present study, to test whether, and if so, how the increased IL-17A expression was responsible for inhibition of Th1 cell function in IBD, we used the human colonic epithelial cell line HT-29 cells, as we have found that the expression of IL-17A in and IL-17R on CEC cells is significantly increased in mice with TNBS-induced colitis, which is an animal model of Crohn’s disease (CD). IL-17A alone had little effect on the activity of HT-29 cells, so we examined its synergistic effects with TNF-α. Treatment of HT-29 cells with IL-17A inhibited the TNF-α-induced increase in expression of mRNAs coding for CXCL11 (Fig. 1B) and IL-12P35 (Fig. 1C), two factors promoting Th1 cell function. We then examined how IL-17A signaling affected the TNF-α-induced activation of CECs. Our data showed that IL-17A signaling enhanced TNF-α induced phosphorylation of ERK (Fig. 1D), AKT (Fig. 1E), and CEBP/β (Fig. 1F). These data show that IL-17A signaling triggers intracellular cascades, which affect TNF-α-induced cytokine production.

**Figure 2:**
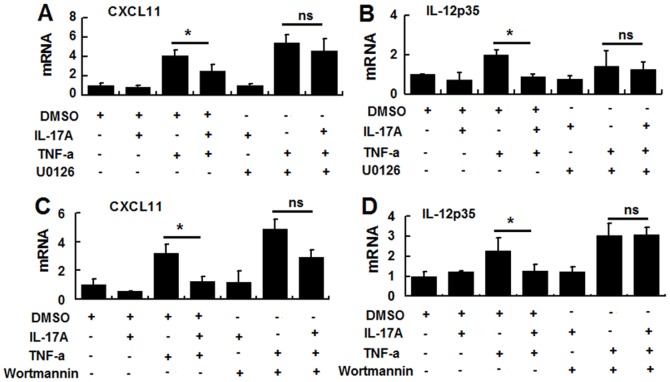
Effects of an ERK or PI3K inhibitor on IL-17A signaling-mediated negative regulation in HT-29 cells. HT-29 cells were incubated with or without an inhibitor specific for ERK(U0126) or PI3K(wortmannin) or DMSO (vehicle control) for 30 min, then IL-17A and/or TNF-α was added and the cells incubated for 6 h in the continued presence of the inhibitor. The cells were then examined for CXCL11 and IL-12P35 expression by real-time PCR. The results shown are representative of those obtained in three independent experiments. The bars are the SD.

To further characterize the intracellular cascades involved in IL-17A-induced negative regulation of TNFα-induced CXCL11 and IL-12P35 mRNA expression, specific inhibitors of ERK (U0126) or PI3K-AKT (wortmannin) were added for 30 minutes before and during cytokine treatment. As shown in Fig. 2, blockade of either ERK or PI3K blocked the inhibitory effect of IL-17A on TNF-α-induced CXCL11 or IL-12P35 mRNA expression. These data show that the ERK and PI3K-AKT pathways play essential roles in IL-17A-mediated negative regulation. We did not examine the effects of CEBP/β blockade on IL-17A mediated negative regulation, as no inhibitor is currently available.

### Act1 is involved in the IL-17A-induced enhancement of the TNF-α-induced phosphorylation of ERK and AKT, and Act1 knockdown prevents IL-17A-induced inhibition of the TNF-α-induced increase in CXCL11 and IL-12P35 mRNA expression

**Figure 3:**
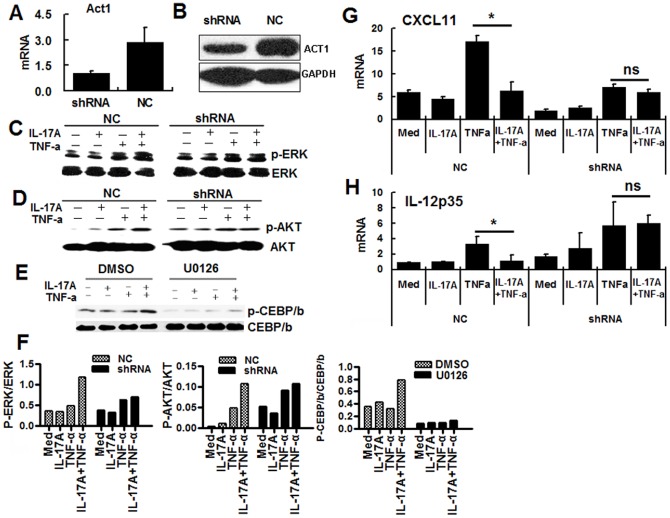
Roles of Act1 in IL-17A-mediated negative regulation in HT-29 cells. (A and B) An Act1 stable knockdown HT-29 cell line was established as described in the Materials and Methods and silencing of Act1 confirmed by real-time PCR (A) and Western blotting (B). (C and D) Act1 knock down or control HT-29 cells were treated with IL-17A and/or TNF-α for 15 min, then cells were examined for phosphorylation of ERK (C) or PI3K-AKT (D) by Western blotting. (E) HT-29 cells were treated with IL-17A and/or TNF-α for 15 min in the presence or absence of the ERK inhibitor, U026, then were lysed and examined for the phosphorylation of CEBP/β. The band intensity data for above western blot assay were shown in F. (G and H) Act1 knock down or control HT-29 cells were treated with IL-17A and/or TNF-α for 6 h, then were examined for levels of mRNAs for CXCL11 (G) or IL-12P35 (H) by real-time PCR. The results shown are representative of those obtained in three independent experiments. The bars are the SD.

Act1 (an activator of NF-κB) is an essential adaptor molecule in IL-17 signaling [19]. To examine whether Act1 was also involved in IL-17A-mediated negative regulation in CECs, Act1 stable knock down HT-29 cells were established. Silencing of Act1 led to decreased expression of Act1 at both the mRNA (Fig. 3A) and protein (Fig. 3B) level. In Act1 knockdown cells, IL-17A signaling failed to enhance TNF-induced phosphorylation of ERK (Fig. 3C) and AKT (Fig. 3D), showing that Act1 is involved in the IL-17A-induced phosphorylation of ERK and AKT. In contrast, Act1 knockdown did not significantly affect IL-17A-induced phosphorylation of CEBP/β (data not shown), suggesting that CEBP/β might be regulated by multiple signaling cascades. However, when HT-29 cells were incubated with the ERK inhibitor U026, IL-17A signaling failed to enhance the TNF-induced phosphorylation of CEBP/β(Fig. 3E), indicating that ERK is an upstream activator of CEBP/β.The band intensity analysis data clearly showed that Act1 is involved in the IL-17A-induced phosphorylation of ERK and AKT, and that ERK plays a role in IL-17A enhanced TNF-a induced phosphorylation of CEBP/β (Fig. 3F). Finally, the effects of Act1 knockdown on IL-17A-mediated negative regulation were examined and the data showed that Act1 knockdown blocked IL-17A-induced inhibition of TNFα-induced increase in CXCL11 (Fig. 3G) and IL-12P35 (Fig.3H) mRNA expression. These data show that Act1 is involved in IL-17A-induced enhancement of TNF-α-induced phosphorylation of ERK and PI3K-AKT and for IL-17A-mediated negative regulation.

### Act1 knockdown decreases the expression of PI3K-cat-gamma and identifies a new pathway (IL-17A-Act1-PI3KIB-AKT) of IL-17A-mediated negative regulation in CECs

**Figure 4:**
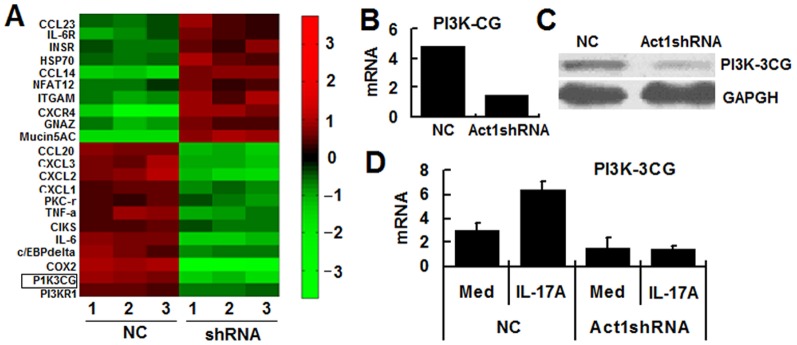
Microarray assay identifies involvement of an Act1--PI3K IB subunit (PI3K-cat gamma) pathway in IL-17A-mediated signaling cascades. (A) Gene chip assay identifies multiple genes differentially expressed in Act1 knock down and control HT-29 cells. (B and C) Act1 knock down decreases PI3K-cat gamma expression as shown by real-time PCR (B) and Western blotting (C). (D) Act1 knock down and control HT-29 cells were treated with recombinant IL-17A for 6 h, then PI3K-cat gamma expression was examined by real-time PCR. The results shown are representative of those obtained in three independent experiments. The bars are the SD.

To investigate the mechanisms by which IL-17A induced negative regulation, microarray analysis was carried out. About 200 differentially expressed genes were present in the knockdown line compared to controls. Of these, expression of chemokines, such as CXCL1 and CXCL2, and cytokines, such as TNF-α, was found to be decreased by more than two-fold in Act1 knockdown HT-29 cells compared to control cells (Fig. 4A); these genes covered a wide range of cellular functions, such as macrophage recruitment. However, we were intrigued by the unexpected finding that PI3K-cat gamma (one subunit of PI3K- IB) expression was more than two-fold lower in Act1 knockdown HT-29 cells and this was confirmed by real-time PCR (Fig. 4B) and Western blotting (Fig. 4C). Notably, we found that IL-17A signaling in the absence of TNF-α increased PI3K-CG expression in control HT-29 cells, but not in Act1 knockdown cells. These data suggest that IL-17A signaling might induce phosphorylation of AKT by increasing PI3K-CG expression, a process dependent on Act1.

### IL-17A negatively regulates Th1 cell activity in a human CEC and PBMC co-culture system

**Figure 5:**
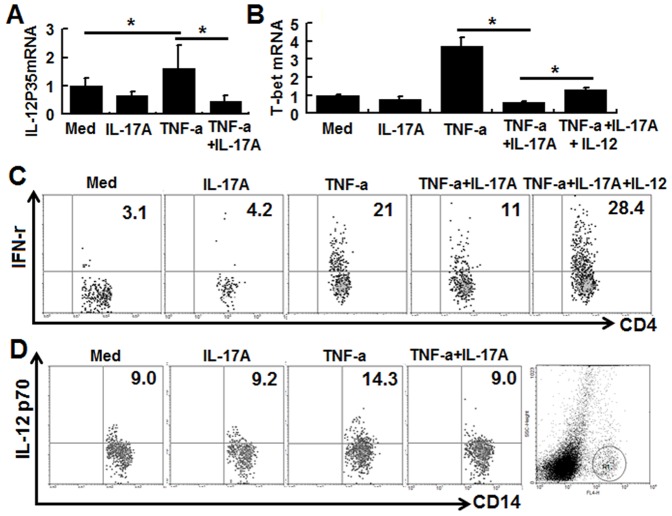
IL-17A signaling mediates negative regulation in a PBMC/HT-29 cell co-culture system. HT-29 cells were cultured in the presence of IL-17A and/or TNF-αfor 24 h, then human PBMCs were added and stimulated with anti-human CD3 and CD28 antibodies with or without recombinant IL-12 for another 24 h. Adherent HT-29 cells were analyzed for IL-12P35 mRNA (A) and non-adherent PBMCs were analyzed for T-bet (B) expression by real-time PCR. IFN-γ expressions within CD4^+^T cells (C) and IL-12P70 expressions within CD14+monocytes (D) were examined by flow cytometry analysis. The results shown are representative of those obtained in three independent experiments. The bars are the SD.

The above data demonstrated that IL-17A signaling inhibits TNF-α-induced mRNA expression of CXCL11 and IL-12P35. To further explore the possible effects of IL-17A signaling, we used an HT-29 cell and human PBMC co-culture system with or without addition of IL-17A. Firstly, human PBMCs were stimulated with anti-human CD3 and CD28 antibodies in the absence or presence of IL-17A and/or TNF-α. We found that recombinant IL-17A did not significantly affect the expression of IL-12P35 mRNA induced by TNF-α (data not shown). Secondly, HT-29 cells were incubated in the presence of IL-17A and/or TNF-α for 24 h, then human PBMCs were added and stimulated with anti-human CD3 and CD28 antibodies for another 24 h, then the non-adherent human PBMCs and adherent HT-29 cells were collected separately and analyzed for gene expression. Our data showed that TNF-α-induced IL-12P35 expression in the isolated adherent HT-29 cells was inhibited by IL-17A (Fig. 5A). And that expression of T-bet, a Th1 cell transcriptional factor, in the non-adherent PBMCs was significantly downregulated in the IL-17A/TNF-α-treated group compared to the TNF-α-treated group, a phenomenon can be reversed by adding recombinant IL-12 p70(Fig. 5B). Flow cytometry analysis examining the IFN-γ expression within CD4+T cells showed the same tendency as that of T-bet (Fig. 5C). These data indicated that IL-17A signaling on HT-29 cells inhibited TNF-α induced Th1 cells function in the co-culture system, in which IL-12 plays an important role. It is known that bioactive form of IL-12 is IL-12 p70 (hetero dimer with p40 and p35). As there is no detectable IL-12P70 secretion in the supernatant and there is no detectable IL-12p35 protein expression within adherent HT-29 cells, the possible source of IL-12 protein were then investigated. Our data showed that IL-17A inhibited TNF-α induced IL-12 protein expression (p70) by CD14+monocytes in the co-culture system (Fig. 5D). These in vitro data again indicated that IL-17A signaling on HT-29 cells may indirectly affect Th1 cell activity by altering the IL-12 expression by monocytes. However, the underlying mechanisms by which IL-17A negatively regulates Th1 cell activity in a human CEC and PBMC co-culture system remain to be investigated.

### IL-17A blockade in vivo leads to exacerbated TNBS colitis and enhanced Th1 related gene/protein expression

**Figure 6:**
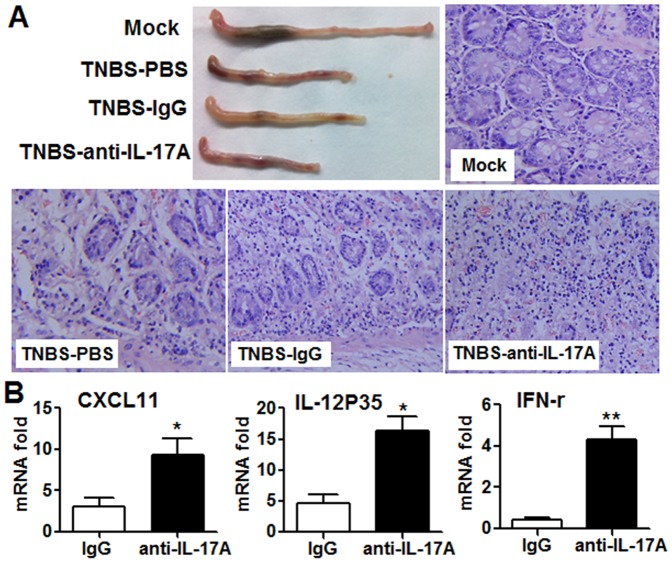
IL-17A blockade in vivo leads to exacerbated TNBS colitis and enhanced Th1 activity. (A-C) The TNBS-colitis model was established in C57BL/6 mice as described in the Materials and Methods and 100 ug of IL-17A neutralizing antibody or control IgG was injected i.p on days 1, 3, 5, and 7 (day 1 is the first day TNBS was administered in the drinking water). Mice were sacrificed on day 8 and examined for tissue damage (A) and CECs (B) isolated from the treated mice were analyzed for CXCL11, IL-12P35, and IFN-γ expression by real-time PCR. The results shown are representative of those obtained in three independent experiments using 8 mice per group. The bars are the SD.

To further examine the axis by which IL-17 mediates negative regulation through CEC cells, in vivo IL-17A neutralization was performed by injection of anti-IL-17A antibody on days 1, 3, 5, and 7 during induction of TNBS-induced colitis and the effects on the activity of CECs examined. Physical and histopathological examination of colon tissue revealed marked tissue injury and infiltration of inflammatory cells in TNBS colitis mice receiving anti-IL-17A antibody (Fig. 6A). IL-17A neutralization enhanced the mRNA expression of CXCL11, IL-12P35, and IFN-γ in splenocytes CECs (data not shown), indicating that neutralization of IL-17A in CD can systemically affect the activity of Th1 cells. It is worthy to note that IL-17A neutralization also enhanced the mRNA expression of CXCL11, IL-12P35, and IFN-γ in CECs (Fig. 6B), showing that CECs are important target for IL-17A mediated regulatory effects.

### Adoptive transfer of CECs derived from TNBS-induced mice exacerbates colitis in mice, which can be inhibited by co-transfer of IL-17

**Figure 7:**
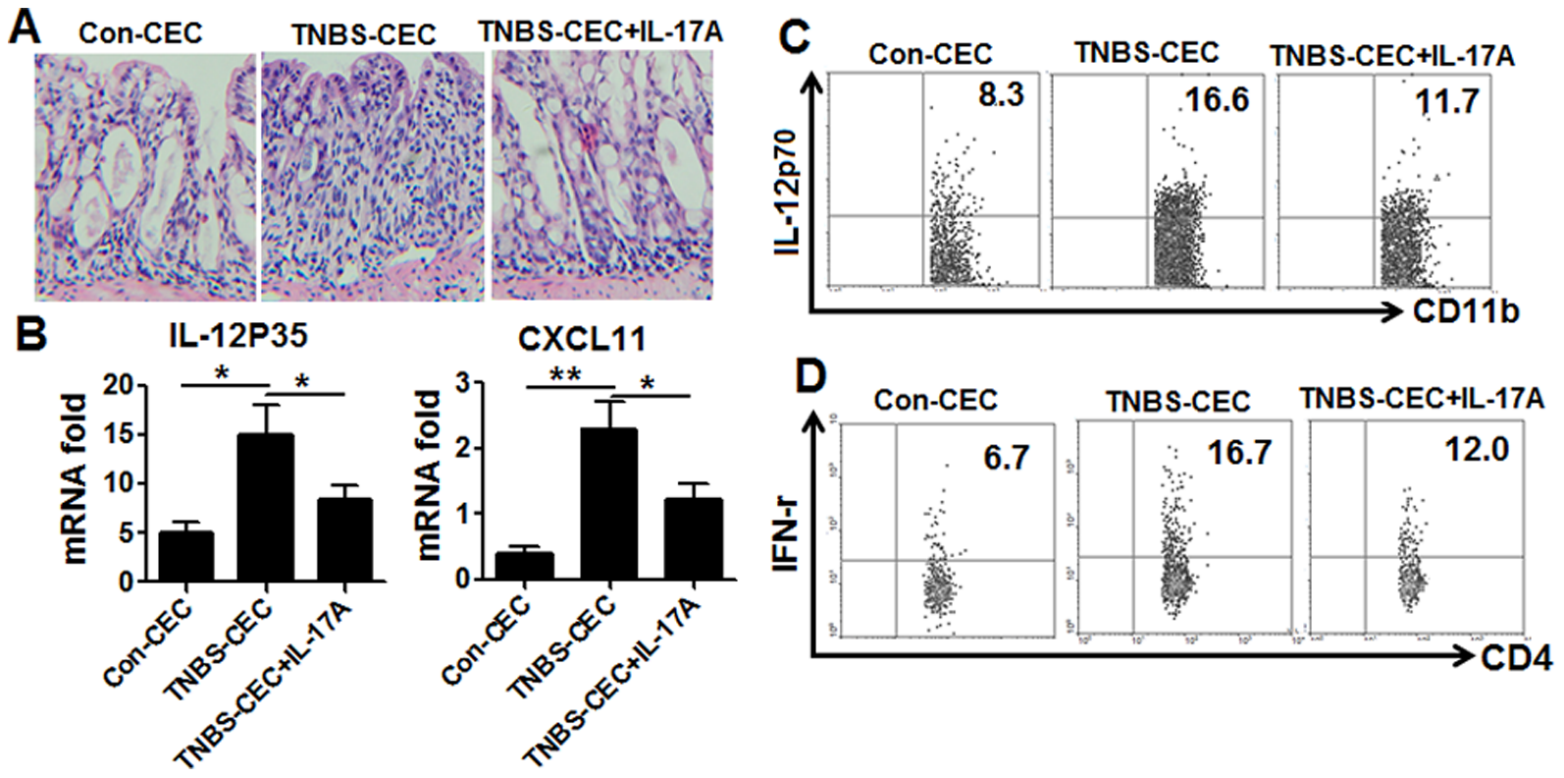
Adoptive transfer of CECs from TNBS-induced mice exacerbates colitis in mice, which can be inhibited by co-transfer of IL-17. CECs were collected from untreated mice (control CECs) or from mice with TNBS-induced colitis on day 8 of colitis induction (TNBS-CEC) and adoptively transferred into TNBS-induced mice (i.p, 1×10^6^/mice) on days 1 and day 4 (TNBS treatment was started on day 1). On day 8, the mice were sacrificed and colon tissue collected for H&E staining (A), CECs were tested for IL-12P35 and CXCL11 mRNA levels by real-time PCR (B). Lymphocytes from colonic lamina propria cells were collected and expressions of IL-12P70 were examined within CD11b^+^ macrophage (C), expressions of IFN-γ were examined within CD4^+^T cells (D). The results shown are representative of those obtained in three independent experiments, each using 6 mice per group. The bars are the SD.

Finally, CECs isolated from mice on day 8 of TNBS-induced colitis were transferred alone or together with recombinant IL-17A into previously untreated mice on days 1 and 4 of induction of TNBS-induced colitis to examine 1) possible roles of CECs in the pathogenesis of CD and 2) whether IL-17A can trigger anti-inflammatory mechanisms in CECs, thus blocking their pathogenic roles in vivo. Adoptively transferred CECs from TNBS-induced colitis mice exacerbated tissue damage (Fig. 7A) and led to increased mRNA expression of CXCL11, IL-12P35, and IFN-γmRNA by CECs of the recipient mice of TNBS colitis mice (Fig. 7B). In addition, transfer of CECs from colitogenic mice into mice without TNBS treatment is associated with an increase of Th1-related cytokines in comparison to mice transferred with CECs from non colitogenic mice (data not shown here). These data showed that CECs from colitogenic mice may affect the Th1 cell activity in vivo after injection. Interestingly, our data clearly showed that administration of IL-17A attenuated the ability of CECs from TNBS-induced colitis mice to induce colitis when transferred into recipients and decreased the expression of CXCL11, IL-12P35, and IFN-γ (Fig. 7B). To further investigate whether and how co administration of IL-17A with CECs affect Th1 cell activity in vivo, we firstly cultured colon tissues and found that colon tissues from TNBS-CECs injected mice produced more IL-12 and IFN-γ than those from Con-CECs injected controls, while co-administration of IL-17A with TNBS-CECs leads to decreased IL-12 and IFN-γ production (data not shown). Secondly, we isolated lamina propria cells and examined the expression of IL-12P70 by CD11b+F4/80+macrophage and of IFN-γ expression by CD4^+^T cells. Our data showed that transfer of CECs alone increased IL-12p70 expression by CD11b+F4/80+macrophage from lamina propria cells. However, co administration of IL-17A with CECs reversed CECs transfer increased IL-12p70 expression by macrophage (Fig.7C). Co-administration of IL-17A lead to decreased IFN-γ expression within CD4^+^T cells (Fig.7D).These data suggested that TNBS-CECs injection with or without IL-17A affected local Th1 response, in which IL-12 might play an important role. Finally, we also examined how IL-17A signaling on CECs, following CECs and IL-17A i.p.injection, affect local Th1 response.

## Discussion

IL-17A plays both pathogenic and protective roles in the progress of IBDs, but the mechanisms by which it mediates its protective effects remain largely unclear [27]–[29]. Here, we demonstrated that IL-17A signaling enhances the TNF-α-induced phosphorylation of the Act1-PI3K (IB)-AKT and Act1-ERK-CEBP/β pathways in CECs, finally inhibiting TNF-α-induced CXCL11 and IL-12P35 mRNA expression. Studies using our in vitro co-culture system and CEC adoptive transfer clearly demonstrated that IL-17A can act on CECs and trigger anti-inflammatory mechanisms against Th1 cells, thus contributing to colonic homeostasis.

Here CECs were selected as the target for IL-17A, as we previously found that, in mice with TNBS-induced colitis, expression of IL-17A in and IL-17RA on CECs was significantly increased (Fig.1A). Although the mechanisms for up-regulating IL-17A and IL-17R expression on CECs following CD remain to be determined, these data indicates that IL-17A/IL-17R pathway might be involved in the physiopathology of IBD. In addition, many reports suggest that, in inflammatory situations, CECs may also act as antigen-presenting cells in the local colonic immune response [30]-[31]. Here, we used a human CEC cell line, HT-29 cell, to investigate the mechanisms by which IL-17A mediated an anti-inflammatory response in CECs. This is the first report showing that IL-17A signaling inhibits the TNF-α-induced increase in IL-12P35 mRNA expression by CECs. Here CXCL11 is selected as it is reported that CXCL11 showed potent activity on activated T cells through selective high affinity binding to CXCR3 which is especially expressed on Th1 cells but not on Th2 cells [32]-[33]. And as an IFN-γ inducible chemokine, the effects of CXCL11 on Th1 cells can be amplified by IFN-γ, a Th1-related cytokine, as a positive feedback [33].

The biologic activity of IL-17A is dependent on a complex composed of IL-17RA and IL-17RC [34]. Here we did not investigate the roles of IL-17A receptor in IL-17A mediated anti-inflammatory effects. In fact, although there are many different reports demonstrating the oppose function of IL-17A [18], [27]-[29], [35], the roles of IL-17A receptor in IL-17A mediated pro-inflammatory and anti-inflammatory effects remain largely unclear.

We then focus on the intracellular mechanisms by which IL-17A signaling inhibits the TNF-a induced expression of IL-12 and CXCL11 by HT-29 cells. We first examined whether NF-κB pathway was involved in IL-17A mediated anti-inflammatory effects in CECs. However, our data showed that IL-17A signaling does not significantly affect the activity of NF-κB, nor it affects TNF-α induced activation of NF-κB (data not shown). So we then focus our manuscript on the MAPK/PI3K pathways. Although it has been reported that the P38 pathway is involved in the IL-17A-mediated pro-inflammatory response [16], we here demonstrated that P38 pathway were not involved in the IL-17A mediated anti-inflammatory response (CXCL11 and IL-12P35 inhibition) ( data not shown). However, IL-17A signaling significantly enhanced TNF-α- induced phosphorylation of ERK in HT-29 cells (Fig. 1). In addition, we also demonstrated the involvement of PI3K-AKT pathway in IL-17A-mediated negative regulation (Fig.2).

Act1 (transcription factor NF-κ B activator 1) is an important adaptor protein in IL-17 receptor (IL-17R) signaling and IL-17A-dependent immune responses [36]. The facts that Act1 expression is increased in colon epithelial cells in mice with IBD and Act1-deficient mice show a delayed onset and much lower severity of DSS-induced colitis [19] suggest that Act1 is involved in the regulation of IBD, but whether or how it is involved in IL-17A-mediated negative regulation remained to be investigated. Our data showing that Act1 knockdown decreased IL-17A-induced enhancement of TNF-α-induced ERK and AKT phosphorylation and blocked IL-17A-mediated negative regulation demonstrate that Act1 plays an essential role in transducing the negative signal of IL-17A in CECs.

Previous report showed that PI3K pathway is involved in IL-17A signaling mainly in an Act1-independent manner [21]. However, here we found that Act1 knock down significantly lead to decreased expression of PI3K- cat gamma 1B (PI3K- 1B) in response to IL-17A stimulation (Fig.4). These data partially explains how Act1 knock down leads to decreased phosphorylation of AKT, and indicates that PI3K pathway might be involved in IL-17A signaling pathway in a manner partially dependent on Act1.

However, it was still not known how the enhanced phosphorylation of ERK and PI3K-AKT led to inhibition of CXCL11 and IL-12P35 mRNA expression. To examine this, the transcriptional factors controlling CXCL11 and IL-12P35 mRNA expression were investigated, among which we focus on the function of C/EBPβ. Data suggest that C/EBPβ can bind to the region bp - 444 and - 392 of the IL-12P35 promoter and negatively regulate LPS-induced expression of the IL-12 subunit P35 [37]and that phosphorylation of C/EBPβ decreases its ability to bind to DNA [38]. As shown in Fig. 1, IL-17A signaling enhanced the TNF-α-induced phosphorylation of C/EBPβ, a process inhibited by blockade of the ERK pathway (Fig. 3), suggesting that ERK activation is the upstream signaling cascade accounting for the phosphorylation of C/EBPβ. Our above data showed that Act1 knockdown decreased IL-17A-induced enhancement of TNF-a-induced ERK phosphorylation (Fig.3). In such a scenario, IL-17A signaling activates Act1 and this enhances the TNF-α-induced phosphorylation of ERK, finally leading to phosphorylation of C/EBPβ, while decreases its ability to bind to the CXCL11 and IL-12P35 promoters, leading to decreased CXCL11 and IL-12P35 mRNA expression.

We then further investigated how the enhanced PI3K-AKT phosphorylation contributes to IL-17A mediated negative regulation. One study in HT-29 cells has suggested that inhibition of PI3-K results in induction of NF-κB binding activity [39]. Consistent with this, a mutation that inactivates PI3Kγ enzymatic activity (‘kinase-dead’) leads to less severe colitis in mice, which produce significantly more pro-inflammatory Th1 cytokines, such as IL-12, TNF-α, and IFN-γ. This suggests a role for PI3Kγ in the negative regulation of these cytokines [40]. In our study, IL-17A signaling alone did not markedly affect TNF-α-induced NF- κB phosphorylation, but wortmannin, a PI3K inhibitor enhanced this process (data not shown), suggesting that IL-17A may inhibit TNF-α-induced NF-γ B phosphorylation by increasing the phosphorylation of PI3K-AKT, although the underlying mechanism remains to be determined.

Whether and how IL-17A-mediated negative regulation affected the local immune response was then investigated. Our co-culture system clearly showed that IL-17A signaling in CECs inhibited the TNF-α-induced increase in IL-12P35 mRNA expression by adherent HT-29 cells, which led to inhibited Th1 cell function, suggesting that IL-17A signaling in CECs can affect the activity of Th cells (Fig.5B&C). Interestingly, our data showed that IL-17A signaling enhanced TNF-a induced IL-12p35 mRNA expression but not protein expression, while IL-17A signaling enhanced TNF-a induced IL-12p70 protein expression by monocytes in the co-culture system, indicating that IL-17A signaling on CECs may affect Th1 cell activity indirectly. A previous report which showed that IL-12 expressing epithelia cells (at mRNA level) promotes the Th1 cell response support our findings [41]. However, the underlying mechanisms by which IL-17A negatively regulates Th1 cell activity in a human CEC and PBMC co-culture system remain to be investigated. In addition, we blocked IL-17A in mice with TNBS- induced colitis in vivo and found that this enhanced CXCL11 and IL-12P35 mRNA expression by CECs. This is the first report demonstrating a negative regulation mechanism of IL-17A on CEC in vivo.

The above data indicate that CECs act as important mediators in the pathogenesis or regulation of IBD, which are consistent with previous reports [42]–[43]. To further demonstrate that CECs were a critical target of IL-17A-mediated negative regulation in vivo, we transferred CECs or co-transferred CECs and IL-17A into TNBS colitis mice. As shown in Fig. 7, transfer of CECs from TNBS colitis mice exacerbated colitis and increased the activity of Th1 cells in recipient mice, while co-transfer of these cells and IL-17A inhibited colitis by inhibiting Th1 cell function in recipient mice further demonstrating that CECs are critical target cells in IL-17A-mediated negative regulation.

In summary, we have demonstrated a regulatory mechanism of IL-17A in the progression of CD. By activating the Act1-ERK-CEBP/β and Act1-PI3K-AKT pathways in CECs, IL-17A signaling negatively regulates TNF-α-induced mRNA expression of CXCL11 and IL-12P35. Our in vivo assay also demonstrated the existence of an IL-17A-CEC- Th1 inhibition axis in IBD. Further investigation of this pathway will shed new light on the pathogenesis and regulation of IBD.

## References

[1] Strober W, Fuss I, Mannon P (2007) The fundamental basis of inflammatory bowel disease. J Clin Invest 117: 514–521. 17332878 10.1172/JCI30587 PMC1804356

[2] Kaser A, Zeissig S, Blumberg RS (2010) Inflammatory bowel disease. Annu. Rev. Immunol 28: 573–621. 10.1146/annurev-immunol-030409-101225 PMC4620040 20192811

[3] Romagnani S (1999) Th1/Th2 cells Inflamm Bowel Dis. 5(4): 285–294. 10.1097/00054725-199911000-00009 10579123

[4] Funakoshi K, Sugimura K, Sasakawa T, Bannai H, Anezaki K, et al (1995) Study of cytokines in ulcerative colitis. . J Gastroenterol. 30 Suppl 861–63. 8563893

[5] Schreiber S, Rutgeerts P, Fedorak RN, Khaliq-Kareemi M, Kamm MA, et al (2005) A randomized, placebo-controlled trial of certolizumab pegol (CDP870) for treatment of Crohn's disease. Gastroenterology 3: 807–818. 10.1053/j.gastro.2005.06.064 16143120

[6] Rahimi R, Nikfar S, Abdollahi M (2007) Do anti-tumor necrosis factors induce response and remission in patients with acute refractory Crohn's disease? A systematic meta-analysis of controlled clinical trials. . Biomed. Pharmacother. 1: 75–80. 10.1016/j.biopha.2006.06.022 17184965

[7] Park H, Li Z, Yang XO, Chang SH, Nurieva R, et al (2005) A distinct lineage of CD4 T cells regulates tissue inflammation by producing interleukin 17. Nat Immunol 6: 1133–1141. 16200068 10.1038/ni1261 PMC1618871

[8] Jovanovic DV, Di Battista JA, Martel-Pelletier J, Jolicoeur FC, He Y, et al (1998) IL-17 stimulates the production and expression of proinflammatory cytokines, IL-1beta and TNF-alpha, by human macrophages. . J. Immunol. 160: 3513–3521. 9531313

[9] Fossiez F, Djossou O, Chomarat P, Flores-Romo L, Ait-Yahia S, et al (1996) T cell interleukin-17 induces stromal cells to produce proinflammatory and hematopoietic cytokines. . J. Exp. Med. 183: 2593–2603. 8676080 10.1084/jem.183.6.2593 PMC2192621

[10] Awane M, Andres PG, Li DJ, Reinecker HC (1999) NF-kappa B-inducing kinase is a common mediator of IL-17-, TNF-alpha-, and IL-1 beta-induced chemokine promoter activation in intestinal epithelial cells. . J. Immunol. 162: 5337–5344. 10228009

[11] Onishi RM, Gaffen SL (2010) Interleukin-17 and its target genes: mechanisms of interleukin-17 function in disease. Immunology 129: 311–321. 20409152 10.1111/j.1365-2567.2009.03240.x PMC2826676

[12] Hueber W, Pate DD, Dryja T, Wright AM, Koroleva I, et al (2010) Effects of AIN457, a fully human antibody to interleukin-17A, on psoriasis, rheumatoid arthritis, and uveitis. . Sci. Transl. Med. 2: 52ra72. 10.1126/scitranslmed.3001107 20926833

[13] Fujino S, Andoh A, Bamba S, Ogawa A, Hata K, et al (2003) Increased expression of interleukin 17 in inflammatory bowel disease. Gut 52: 65–70. 12477762 10.1136/gut.52.1.65 PMC1773503

[14] Rovedatti L, Kudo T, Biancheri P, Sarra M, Knowles CH, et al (2009) Differential regulation of interleukin 17 and interferon gamma production in inflammatory bowel disease. Gut 58: 1629–1636. 19740775 10.1136/gut.2009.182170

[15] Hueber W, Sands BE, Lewitzky S, Vandemeulebroecke M, Reinisch W, et al (2012) Secukinumab, a human anti-IL-17A monoclonal antibody, for moderate to severe Crohn's disease: unexpected results of a randomised, double-blind placebo-controlled trial. . Gut. 61(12): 1693–1700. 22595313 10.1136/gutjnl-2011-301668 PMC4902107

[16] Lee JW, Wang P, Kattah MG, Youssef S, Steinman L, et al (2008) Differential regulation of chemokines by IL-17 in colonic epithelial cells. . J. Immunol. 181: 6536–6545. 18941244 10.4049/jimmunol.181.9.6536

[17] Liang SC, Tan XY, Luxenberg DP, Karim R, Dunussi-Joannopoulos K, et al (2006) Interleukin (IL)-22 and IL-17 are coexpressed by Th17 cells and cooperatively enhance expression of antimicrobial peptides. . J. Exp. Med. 203: 2271–2279. 16982811 10.1084/jem.20061308 PMC2118116

[18] O'Connor W, Kamanaka M, Booth CJ, Town T, Nakae S, et al (2009) A protective function for interleukin 17A in T cell-mediated intestinal inflammation. Nat. Immunol. 10: 603–609. 19448631 10.1038/ni.1736 PMC2709990

[19] Qian Y, Liu C, Hartupee J, Altuntas CZ, Gulen MF, et al (2007) The adaptor Act1 is required for interleukin 17-dependent signaling associated with autoimmune and inflammatory disease. Nat. Immunol. 8: 247–256. 17277779 10.1038/ni1439

[20] Ruddy MJ, Wong GC, Liu XK, Yamamoto H, Kasayama S, et al (2004) Functional cooperation between interleukin-17 and tumor necrosis factor-α is mediated by CCAAT/enhancer binding protein family members. . J. Biol.Chem. 279: 2559–2567. 14600152 10.1074/jbc.M308809200

[21] Huang F, Kao CY, Wachi S, Thai P, Ryu J, et al ( 2007) Requirement for both JAK-mediated PI3K signaling and ACT1/TRAF6/TAK1-dependent NF-kappaB activation by IL-17A in enhancing cytokine expression in human airway epithelial cells. J. Immunol. 179: 6504–6513. 17982039 10.4049/jimmunol.179.10.6504

[22] Shi F, Guo X, Jiang X, Chen G, Zhou P, et al ( 2012) Dysregulated Tim-3 expression and its correlation with imbalanced CD4 helper T cell function in ulcerative colitis. Clinical. . Immunology. 145: 230–240. 10.1016/j.clim.2012.09.001 23117395

[23] Lytle C, Tod TJ, Vo KT, Lee JW, Atkinson RW, et al (2005) The peroxisome proliferator-activated receptor γ ligand rosiglitazone delays the onset of inflammatory bowel disease in mice with interleukin 10 deficiency. . Inflamm. Bowel. Dis. 11: 231–243. 15735429 10.1097/01.mib.0000160805.46235.eb

[24] Neurath MF, Fuss I, Kelsall BL, StiJber ER, Strober W (1995) Antibodies to IL-12 abrogate established experimental colitis in mice. J. Exp. Med. 182: 1281–1290. 7595199 10.1084/jem.182.5.1281 PMC2192205

[25] Li X, Chen G, Li Y, Wang R, Wang L, et al (2010) Involvement of T Cell Ig Mucin-3 (Tim-3) in the Negative Regulation of Inflammatory bowel diseases. . Clin Immunol. 134: 169–177 19913460 10.1016/j.clim.2009.09.012

[26] Lefrançois L, Lycke N (2001) Isolation of mouse small intestinal, intraepithelial lymphocytes, Peyer’s Patch, and lamina propria cells. Curr. Protoc. Immunol.3.19.1-3.19.16. 10.1002/0471142735.im0319s17 18432783

[27] Ito R, Kita M, Shin-Ya M, Kishida T, Urano A, et al (2008) Involvement of IL-17A in the pathogenesis of DSS-induced colitis in mice. . Biochem. Biophys. Res. Commun. 377: 12–16. 18796297 10.1016/j.bbrc.2008.09.019

[28] Ogawa A, Andoh A, Araki Y, Bamba T, Fujiyama Y (2004) Neutralization of interleukin-17 aggravates dextran sulfate sodium-induced colitis in mice. . Clin. Immunol. 110: 55–62. 14962796 10.1016/j.clim.2003.09.013

[29] Yang XO, Chang SH, Park H, Nurieva R, Shah B, et al (2008) Regulation of inflammatory responses by IL-17F. . J. Exp. Med. 205: 1063–1075. 18411338 10.1084/jem.20071978 PMC2373839

[30] Pallone F, Fais S, Capobianchi MR (1988) HLA-D region antigens on isolated human colonic epithelial cells: enhanced expression in inflammatory bowel disease and in vitro induction by different stimuli. . Clin. Exp. Immunol. 74: 75–79. 3146453 PMC1541709

[31] Hansen JJ, Holt L, Sartor RB (2009) Gene expression patterns in experimental colitis in IL-10 deficient mice. Inflamm. . Bowel. Dis. 15: 890–899. 10.1002/ibd.20850 PMC2763592 19133689

[32] Bonnechi RG, Bianchi PP, Bordignon DD (1998) Differential expression of chemokine receptors and chemotactic responsiveness of type 1 T helper cells (Th1s) and Th2s, J. Exp. Med 187(1): 129–134. 10.1084/jem.187.1.129 PMC2199181 9419219

[33] Cole KE, Strick CA, Paradis TJ, Ogborne KT, Loetscher M, et al (1998) Interferon-inducible T cell alpha chemoattractant (I-TAC): a novel non-ELR CXC chemokine with potent activity on activated T cells through selective high affinity binding to CXCR3, J. Exp. Med 187(12): 2009–2021. 10.1084/jem.187.12.2009 PMC2212354 9625760

[34] Toy D, Kugler D, Wolfson M, Vanden Bos T, Gurgel J, et al (2006) Cutting edge: interleukin 17 signals through a heteromeric receptor complex. . J Immunol. 177(1): 36–39. 16785495 10.4049/jimmunol.177.1.36

[35] Yamada S, Naito Y, Takagi T, Mizushima K, Hirai Y, et al (2011) Reduced small-intestinal injury induced by indomethacin in interleukin-17A-deficient mice. . J Gastroenterol Hepatol. 26(2): 398–404. 21261732 10.1111/j.1440-1746.2010.06496.x

[36] Gaffen SL (2009) Structure and signalling in the IL-17 receptor family. Nat. Rev. Immunol. 9: 556–576. 19575028 10.1038/nri2586 PMC2821718

[37] Kollet J, Witek C, Gentry JD, Liu X, Schwartzbach SD, et al (2001) Deletional analysis of the murine IL-12 p35 promoter comparing IFN- g and lipopolysaccharide stimulation. . J. Immunol. 167: 5653–5663. 11698437 10.4049/jimmunol.167.10.5653

[38] Shen F, Li N, Gade P, Kalvakolanu DV, Weibley T, et al (2009) IL-17 receptor signaling inhibits C/EBPbeta by sequential phosphorylation of the regulatory 2 domain. . Sci. Signal. 2 ra8. 19244213 10.1126/scisignal.2000066 PMC2754870

[39] Wang Q, Kim S, Wang X, Evers BM (2000) Activation of NF-κB binding in HT-29 colon cancer cells by inhibition of phosphatidylinositol 3-kinase. Biochem. . Biophys. Res. Commun. 273: 853–858. 10.1006/bbrc.2000.3034 10891336

[40] van Dop WA, Marengo S, te Velde AA, Ciraolo E, Franco I, et al (2010) The absence of functional PI3Kγ prevents leukocyte recruitment and ameliorates TNBS-induced colitis in mice. . Immunol. Lett. 131: 33–39. 20347874 10.1016/j.imlet.2010.03.008

[41] Ueno N, Fujiya M, Segawa S, Nata T, Moriichi K, et al (2011) Heat-killed Body of Lactobacillus brevis SBC8803 Ameliorates Intestinal Injury in a Murine Model of Colitis by Enhancing the Intestinal Barrier Function. Inflamm Bowel Dis 17: 2235–2250. 21987297 10.1002/ibd.21597

[42] Pallone F, Fais S, Capobianchi MR (1988) HLA-D region antigens on isolated human colonic epithelial cells: enhanced expression in inflammatory bowel disease and in vitro induction by different stimuli, Clin Exp Immunol. 74(1): 75–79. PMC1541709 3146453

[43] Brandtzaeg P, Halstensen TS, Huitfeldt HS, Krajci P, Kvale D, et al (1992) Epithelial expression of HLA, secretory component (poly-Ig receptor), and adhesion molecules in the human alimentary tract. . Ann N Y Acad Sci. 664: 157–179. 1456647 10.1111/j.1749-6632.1992.tb39758.x

